# On the Uses of Tobacco in Inflammatory Diseases

**Published:** 1852-01

**Authors:** Wm. Matthews

**Affiliations:** Nicholsonville, Indiana


### ARTICLE III.

*On the uses of Tobacco in certain Inflammatory Diseases.* By
WM. MATTHEWS, M. D., Nicholsonville, Indiana.

Direct depletion, by the lancet, in the treatment of the diseases of the West, is almost totally neglected by our physicians. Fèw persons, however robust they may appear, when attacked by inflammatory disease, bear direct depletion to the same extent in the Mississippi Valley that they would, were they located upon the Eastern shores of this great continent. Hence it is, that the most successful practitioners of the West, in the remedial management of inflammatory affections, in conjunction with a moderately depletory mode of treatment, use roborant and tonic medicines, together with a pretty liberal diet. Our atmosphere, we have abundant reason for believing, is never wholly free from the depressing agency of the so called malaria, supposed to be diffused in the air by all low and marshy districts, when long continued heat is brought to bear upon the surface of the earth. The modifying influence of this agent, over pathological conditions, it is, therefore, believed, calls for a corresponding modification in the application of therapeutical remedies. And as has just been stated the experience of practical men of the West, bears us out in such an inference.

Even in pneumonic and peritoneal fevers, we of the Mississippi Valley are taught, and some of us by sad experience, too, to treat by cautious abstractions of the blood, by opiate and mercurial medicines, vesication, and last, though not least, by the liberal and timely employment of quinia, and nutritious soups. I appeal to my medical brethren to bear me out in this statement. I have repeatedly used quinia, under such circumstances, with results the most satisfactory, and I doubt not but that others have done the same thing.

Now, I believe, from considerable experience, and, also, from the testimony of several highly respectable practitioners of my acqnaintance, that we possess an article of the materia medica which may be profitably substituted for direct depletion by the lancet, in nine-tenths of the acute inflammatory diseases of our Western country. That article is the common TOBACCO, to be found in the pockets and mouths of a majority of our physicians, at all times. I do not pretend to say that it should take the place of the lancet in *every* case, but I know that in a vast majority of instances it may be profitably substituted for that instrument. As an arterial sedative in the management of all forms of disease whose focus of irritation, so to speak, is the mucous tissue of the stomach and bowels, it is, without doubt, very greatly to be preferred to the preparations of antimony, which have a well known tendency while they control the momentum of the blood, to aggravate the disturbed condition of these structures. It will, when efficiently employed in such cases, I am well satisfied, fulfill the indication of controlling the febrile movements without, at the same time, harrowing up those delicate membranes upon the physiology of which the welfare of our patients so often depends.

In certain specific inflammations, for example, in which typhoid symptoms are apt to be very early developed, tobacco is capable of exerting an almost magical influence. This is particularly true respecting erysipelas—a disease which for many year's past has been mowing down its thousands throughout the West, and the nature of which is specific inflammation of the throat, with low typhoid complications. In instances of this kind the judicious employment of tobacco has been known to break down the activity of the disease, and leave the patient in a debilitated, though convalescing condition, in the course of a few hours. So salutary are its effects in such cases, that a physician of my acquaintance, who had used it much, remarked to me, that he felt as confident he would cure a case of erysipelas with tobacco, as I did that I would relieve a case of intermittent fever, for which I had just made a prescription of a scruple of quinine. And another said to me, after several years of experience with the article, that it was capable of curing all curable cases.

From a few trials, which I have made with the present article in acute epidemic dysentery, I am persuaded that it is capable of doing much good in its earliest stages. Indeed, I am strongly inclined to the opinion, that erysipelas and dysentery, as these affections have recently appeared in the West, are in their nature closely allied to each other. In the former, the inflammation is principally confined to the fauces, while, in the latter, the large intestine is the structure upon which the intensity of the disease falls. In both, however, we have the same typhoid tendency, and it has repeatedly been the case, that the glands about the neck and arm-pits have suffered inflammation and suppuration, as well in dysentery as in erysipelas. But be this as it may, the chief indication in the remedial management of flux, is to subdue symptomatic fever. If this can be done, and the strength of the patient at the same time can be preserved, little danger need be apprehended. Copious abstraction of blood, it has been supposed, is capable of subduing such arterial excitement, and such is undoubtedly true. But suppose this indication is fulfilled by blood-letting, we need not be surprized if, through the exhausting nature of the disease, and the depressing agency of the remedy, the patient falls into a true typhoid fever, from which he dies, or recovers only after weeks of fearful debility.

Now no such fears need be entertained as to the employment of tobacco. Its depressing effects may be removed at pleasure, and the vital forces allowed to resume their healthy action. The nutrition of the blood is in no wise impaired, and convalescence is, therefore, apt to be rapid and perfect. Nor need there be any fears entertained about carrying the remedy too far. I think we have an unerring guide as to this, and it now remains only to give:

*The mode of its application.*—This is exceedingly simple. The leaves are moistened and spread smoothly out several thicknesses over the abdomen, or throat, or the extremities are covered, or all these parts may be covered at one and the same time. From time to time they should be moistened or sprinkled with warm water, and kept thus applied until the specific effects of the tobacco are obtained. In some instances this may be procured in the course of a few hours, but in others, from twelve to twenty-four hours will be required.

The indications for removing the tobacco are the following: The pulse becomes less frequent and quick, the skin soft, slight narcotism, nausea, vomiting, and not unfrequently, purging. Along with the change thus wrought upon the heart and nerves, the local inflammation is found, in many instances, to have almost entirely given way.

After the removal of the leaves, should re-action take place it may be proper to repeat their application, just as we would repeat venesection under like circumstances.

The decoction may be used, instead of the tobacco in substance, by means of flannel strips. Or, in cases of emergency, it may be employed in the form of enema, observing proper precaution to not prostrate the vital forces too much.

I do not claim the foregoing remarks to be wholly original with me, but presume they will be new to many practitioners. And it is hoped such may profit by them.

